# Honey Bee (*Apis mellifera* L.) Broods: Composition, Technology and Gastronomic Applicability

**DOI:** 10.3390/foods11182750

**Published:** 2022-09-07

**Authors:** Raquel P. F. Guiné, Sofia G. Florença, Paula M. R. Correia, Ofélia Anjos, Catarina Coelho, Cristina A. Costa

**Affiliations:** 1 CERNAS-IPV Research Centre, Polytechnic Institute of Viseu, 3504-510 Viseu, Portugal; 2 Faculty of Food and Nutrition Sciences, University of Porto, 4200-465 Porto, Portugal; 3 School of Agriculture, Polytechnic Institute of Castelo Branco, 6001-909 Castelo Branco, Portugal; 4 Forest Research Centre, School of Agriculture, University of Lisbon, 1349-017 Lisbon, Portugal

**Keywords:** honey bee brood, pupae, larvae, nutritional value, food ingredients, gastronomy

## Abstract

Honey bee broods (larvae and pupae) can be consumed as human food, offering a rich nutritional value. Therefore, the objective of this work was to present an overview of the nutritional value of the honey bee brood and its gastronomic potential. The results indicated that honey bee broods are rich in protein (including essential amino acids), fat (essentially saturated and monounsaturated fatty acids), carbohydrates, vitamin C and those of the B complex, and minerals such as potassium, magnesium, calcium, and phosphorous. The results further highlight some variability according to the stage of development, with increasing content of fat and protein and decreasing carbohydrates from the larval to the pupal stages. The production of the honey bee brood in the hive, as well as its removal, can impact the wellbeing of the hive. This limits the production potential of the brood aimed at application for gastronomic purposes. The consumption and purchase of honey bee broods as food may be accessible in specialised markets where, for example, ethnic communities consume this type of food. However, in some markets, insects or products produced from insects are not readily accepted because of neophobia and disgust. The role of culinary chefs allied to traditional ways of preparing culinary dishes that include honey bee broods are relevant to motivate more people in western societies to consume of these types of food products.

## 1. Introduction

Insects have been demonstrated to be a valuable resource for the future of humankind from different perspectives: food security, sustainability and environmental concerns, or socioeconomic relevance [1]. In this context, many insects and insect-derived products have been suggested for use as foods or food ingredients. Hocking and Matsumura [2] published a reference article about honey bee (*Apis mellifera* L.) broods as food, stating the value and potential for consumption, forms of presentation in the market, and economic considerations.

Honey bee broods (larvae and pupae) are nontoxic and have a very rich nutritional value, presenting a high content of protein and fat similar to beef, but richer in minerals and most vitamins [3]. However, Skinner [4], using high-performance liquid chromatography (HPLC), could not detect retinol (vitamin A) or retinyl palmitate in the larvae and pupae of *Apis mellifera*, thus concluding that these products did not constitute a source of vitamin A for dietary purposes, later confirmed by data reported by Krell [3] and Finke [5].

Jensen et al. [6] described the methodologies that must be used to evaluate the nutritional composition of honey bee broods to be served as human food, including protein and amino acid composition, lipid and fatty acid composition, vitamins (fat soluble and water soluble), and minerals and ash, among other bioactive components such as antioxidants.

Utilising honey bee larvae and pupae for human consumption is a culturally acceptable and regular practice in many countries worldwide, while not considered normal in other cultures. They are included in diverse culinary preparations or used as ingredients, and their processing involves many operations and different cooking methods. Forms of consumption include dried, cooked (ex. fried), and canned, among others. When cooked or dried, they retain their shape and present a pleasantly crunchy texture and an intense nutty flavour [7].

Male honey bee (drone) broods have a high nutritional value, including amino acids (including essential amino acids), fatty acids (including monounsaturated oleic acid), minerals, and vitamins B_3_ and B_5_ [8]. Thus, they offer an alternative source of nutrients for human consumption. Drone broods also have other applications besides being food for humans, namely in medicine, with reported effects on fertility problems, nervous and mental diseases, malnutrition (improving appetite and weight gain), and enhancing immunity against viral diseases [9,10,11].

Insect farming, markets, and commercialisation are experiencing considerable growth, in which the domain of animal feed is undoubtedly a powerful component [1]. The future of insects as human food and animal feed seems promising given the recent trends. Nevertheless, there are several challenges related to using honey bee broods. Such challenges include nutritional, social, technological, environmental, and economic issues.

From a nutritional point of view, the characteristics and quality of honey bee broods are influenced by a set of factors that arise from bees’ nutrition, developmental stages, castes, body weight, and health status [12,13]; and habitat and climate conditions as well as processing and preparation methods [13,14].

In relation to the nutritional content of honey bee broods, despite some papers that have focused on this, larger studies are needed as there is considerable variation associated with insect castes and life stage, collection site (geographical locations and eco-zones), insect feed, rearing technology, and processing method [13]. In addition, little is known about the bioactive compounds in bee broods that might add to the value of this food product [12].

Several studies worldwide reported pesticides in bee wax, honey, bees, and pollen, usually taken from inside the hive [15], but to our knowledge no studies were performed for pesticide detection specifically in bee broods. Only coumaphos, an organophosphate acaricide, was found in bee broods after a *Varroa sp*. treatment [16]. In fact, coumaphos was reported to be present in honey above the established maximum residue limits (MRLs) and it seems to accumulate in the wax year after year [17,18].

Regarding antimicrobial residues, currently European Union regulation No. 37/2010 (Commission Regulation (EU) No. 37/2010) has not established MRLs for antimicrobial substances in honey and therefore the use of antibiotics in beekeeping is not allowed in the European community. Even though antibiotic drugs are not authorised for the treatment of bees, many studies show the presence of residues in honey, raising the suspicion that this is caused mainly by its illegal use in beekeeping [19]. No studies were found on bee broods.

Borkovcová et al. [20] analysed drone broods collected from an industrial area (Opava, in the Czech Republic), and the lead content was considered high (0.21 mg/100 g), but the author thought this heavy metal content could be a consequence of the industrial activity nearby.

Until now, limited work has been done to answer those questions and challenges, and intensive research is needed to support the use of insects as food and feed [13]. Furthermore, only a few species are studied well enough to be reared as domesticated insects [13].

Finally, there is still a need for regulation and legislation for the use of drone broods as feed, in order to support proper production, transformation, and commercialisation and to ensure food and environmental safety [12,13,21,22,23].

From a social point of view, the reduced acceptability of edible insects in western countries [13], together with “westernisation” in terms of food choices or changes in food habits in countries where insects are culturally part of the local diet [24] are also challenges to overcome. Labelling, documenting, and informing might contribute to boosting consumers’ knowledge of and interest in insects as a food choice [13,25].

The facilities for rearing suitable insect species, both industrial or small mass-production units, and development of safe and efficient production systems and safety control systems, where hygiene and sanitation are central points, is a necessity to ensure the safety of the product [12,24,26]. Processing and food safety procedures, including critical biological and chemical points during collection, transformation, and storage, and the shelf life of insects, fresh or processed, also need further research [13,27].

A major issue to overcome in honey bee brood use is the extraction of broods from the wax combs. When fresh, honey bee broods are quite fragile, and can rupture and oxidize easily. To preserve and facilitate the extraction, freezing is a possibility. Nevertheless, the brood and wax defrost very quickly, which limits the amount of material that can be handled at one time and makes it difficult to separate the brood and wax [6,28].

From an environmental perspective, the possibility of contamination with toxic chemicals that result from the use of pesticides used for protection against pests and parasites should be considered (Jensen et al. 2019).

Despite bees being a well-domesticated species worldwide, and that drone brood removal is a sustainable practice to control *Varroa sp.*, it is a complex technique that most beekeepers aren’t familiar with. The introduction of drone brood frames to reduce *Varroa* sp. infestations and produce drone broods needs proper training and a mindset change (from using a chemical to adopting a complex technique that needs precise and on-time interventions and continuous monitoring). In economic terms, the use of drone brood removal instead of chemical substances will reduce pesticide costs and increase revenue for the beekeepers, who will gain a new beehive product [28]. Along with these benefits, it is also important to consider other non-market values based on the health and environmental benefits and ecosystem services provided.

Hence, the purpose of this article is to make an overview of the nutritional value of honey bee broods and also their gastronomic potential, addressing their processing and culinary uses.

## 2. Composition of Honey Bee Broods

An exhaustive depiction of the chemical composition and nutritional value of the bee brood was presented by Finke [5], highlighting its potential for use as human food (Table 1). This work evaluated the proximate composition, energetic value, and content of a wide variety of vitamins and dietary minerals, as well as amino acids (essential and non-essential) and fatty acids (saturated, monounsaturated, and polyunsaturated). The results revealed that bee broods are an abundant source of protein, fat, and carbohydrates, although poor in fibre and ash (Table 1). In a recent review by Rutka et al. [9], some values for the proximate composition of larval and pupal homogenate are presented on a dry basis: 35.3% and 45.9% protein, 14.5% and 16.0% fat, 46.1% and 34.3% carbohydrates, and 4.1% and 3.8% ash for larvae and pupae, respectively. These results show a rise in protein and fat content from the larval to the pupal stages of development while the carbohydrate content diminishes.

The nutritional composition of other edible insects’ pupae is highly variable. Silkworm (*Bombyx mori*) pupae have 21.5% protein content [29], while the silkworm (*Samia ricini*) pupae and mealworm (*Tenebrio molitor*) pupae have 54.8% [30] and 51% [31], respectively. Regarding fat content, silkworm (*Bombyx mori*) pupae have 13% [29], eri silkworm (*Samia ricini*) have 26.2% [30], and mealworm (*Tenebrio molitor*) pupae have 32% [31]. The fibre content of silkworm (*Bombyx mori*) pupae is 14% [32], while the silkworm (*Samia ricini*) is 4.2% [30] and mealworm (*Tenebrio molitor*) pupae is 12% [31]. Chemical composition and nutritional value of edible insects are variable, not only due to a large number of edible insect species, but also because of the differences between different metamorphic development stages [31,32].

Although not rich in calcium, the honey bee brood constitutes a rich source of other macrominerals, for example phosphorus and magnesium. Some of these minerals are linked to health benefits, namely bone health. Magnesium is also involved in a lot of healthy biochemical reactions in the body [35]. The brood is rich in potassium and chloride but low in sodium. Concerning trace minerals, the honey bee brood has considerable amounts of iron, zinc, copper, and selenium while being poor in manganese and iodine. Regarding the amino acid content, the most abundant essential amino acids are leucine and lysine, while the highest amounts of nonessential amino acids are for glutamic acid, aspartic acid, and proline (Table 1). Finke [5] reported a protein recovery in amino acids equal to 86.8% of total nitrogen (including taurine), and the value was even higher if considering ammonia (88.8%).

The results in Table 1 also reveal that honey bee broods do not contain the fat-soluble vitamins A, D, and E, nor beta-carotene or vitamin B_12_. On the other hand, honey bee broods are a good source of vitamin C, choline, and most vitamins of the B group.

The fatty acid profile shows that the two primary fatty acids in honey bee broods are the monounsaturated oleic acid and the saturated palmitic acid. The profile further reveals that most fat corresponds to saturated (51.8%) and monounsaturated (46.2%) fatty acids, with only a small fraction of polyunsaturated fatty acids (2.0%) [5].

Ghosh et al. [36] reported the nutritional value and chemical composition (proximate composition, energy value, amino acids, fatty acids, and minerals) of larvae, pupae, and adults of *Apis mellifera ligustica* worker bees for human consumption. Table 2 presents the results obtained for larvae and pupae, and they show that there are, in general, no expressive differences between the larvae and the pupae, except for some of the components, such as the protein content, which is much higher in the pupae than in the larvae, or the carbohydrates, which are higher in the larvae. These results are similar to what was reported by Rutka et al. [9] for drone brood homogenate. The results by Ghosh et al. [36] (Table 2) confirm that the larvae and pupae have very similar fatty acid and amino acid profiles, as well as mineral contents, but with slightly higher values in the pupae when compared with the larvae (Table 2). The saturated and monounsaturated fatty acids are predominant, as was reported previously by Finke [5].

A more recent work by Haber et al. [37] revealed that some nutritional components of the edible larvae and pupae of honey bees are influenced by their diet (Table 3). When supplemented with sugar, honey bee broods had a higher protein content, fatty acid composition, and antioxidant properties. However, this work had a more limited scope, since the evaluated elements were only the proximate composition and the fatty acid profile. The results follow a similar trend to previously reported results, with a higher protein content in the pupae than in the larvae. Again, the polyunsaturated fatty acids (C18:2 and C18:3) represent a meagre fraction of the fat, both in the pupae and in the larvae, with the oleic (C18:1) and palmitic (C16:0) acids being the most abundant (Table 3).

A review from Ghosh et al. [38] compiled some data for the chemical composition and functional properties at different developmental stages of honey bee workers belonging to different species and drone broods belonging to different subspecies. Table 4 presents the amino acid profiles of drone pupae over different stages (from prepupal to late pupal) whose ranges of values are gathered from results reported by the same authors in previous studies [39,40]. The most abundant essential amino acids are leucine, lysine, and aromatic amino acids. In contrast, the nonessential amino acid present in higher amounts is glutamic acid, and this trend is common to all *Apis mellifera* subspecies.

The same authors [38] presented data for the fatty acid profiles of drone pupae in different developmental stages for several subspecies of *Apis mellifera*, as shown in Table 5. The obtained values were collected from previous works by the same authors [40,41]. Most fatty acids in pupae from *Apis mellifera* correspond to saturated fatty acids (more than 50%) followed by monounsaturated fatty acids and the polyunsaturated fatty acids, which are in minor fractions (only about 1%), this trend being similar regardless of the subspecies. The most abundant fatty acids include palmitic (C16:0), oleic (C18:1), and stearic (C18:0) acids.

Table 6 shows the mineral content of drone pupae of *Apis mellifera* subspecies in different development stages, based on the values reported in several studies [39,40]. It is relevant to note the quantity of minerals such as potassium, phosphorus, magnesium, and calcium in all subspecies of *Apis mellifera*. Minerals are usually obtained from the diet and the variation in brood mineral content is dependent on ecological and environmental conditions [17].

The results in Table 7 are for the vitamin content of honey bee pupae in different developmental stages and are a summary of data reported in other studies [2,5,42,43]. Although the results refer only in some vitamins and in some of the subspecies, vitamin C is present in worker larvae (at day 9) as well as in worker pupae (day 19) and honey bee brood. This last was also analysed in more detail for a number of B complex vitamins.

## 3. Processing and Uses

Although eating insects is considered normal in many areas of the globe where this practice is culturally accepted and valued, it is also true that in other regions of the world, people have developed a solid reluctance to entomophagy [44,45]. Over two billion people worldwide consume insects regularly as part of their traditional diets [46]. The consumption of honey bee broods in particular is also characteristic in several parts of the world, most especially in tropical areas [38]. Drone broods, in particular, could be considered a minor hive product, however not so widespread as other honey bee products. The brood of the honey bee is an up-and-coming edible resource if we take into account that those honey bees are kept by humans worldwide. Ghosh et al. [38] proposed that drone broods have considerable potential for use in human nutrition, either as food or as an ingredient in food preparations.

### 3.1. Production

Honey bee brood production starts when pollen supply increases (usually in spring) [6]. Honey bee brood size is highly variable, with the drones’ brood being produced in small quantities (two hundred to one thousand).

Several factors influence this variability, including the bee breed, the colony size, the amount of honey, the quantity of pollen, and the number of brood combs present in the hive [47]. As workers are necessary in the hive, it is only advisable to consider the removal of drone larvae and pupae, as it has lower effects on colony performance than the removal of worker larvae and pupae. During drone production season, the propensity for worker bees to build fresh drone combs when drone wax frames are positioned inside the hive serve as an encouragement for the queen bee to lay drone eggs [47,48]. In some areas of the world, the removal of brood combs has been used by beekeepers as a strategy to enhance the maintenance of the hive as well as to control the population of the *Varroa destructor* mite [49], a significant parasite that causes major losses in beekeeping worldwide [50,51]. In fact, drone brood removal is considered a non-chemical and sustainable *Varroa* control method [52].

Drone larvae are generally bigger than worker bees’ larvae because they are fed with higher quantities of pollen and honey and might provide a resource to increase the beekeepers’ income, if properly valued [6].

As drones have important mating functions, to ensure the colony’s productivity and survival beekeepers cannot remove all of the drone brood [53,54,55].

Finally, insects offer a way to generate income for small family farms and other intervening agents along the food supply chain. These assume particular importance in low-income countries.

### 3.2. Collection

The collection of honey bee broods should be made before the pupae’s eyes become pink, since after that the chitin amounts will be increased, compromising their organoleptic quality and possible gastronomic utilisation [6].

Some techniques have been reported for the removal of honey bee broods from the comb cells: (a) By shaking the honeycomb with opened or unsealed cells and larvae/pupae being knocked out. It is, however, necessary to be careful not to break the comb, which should have been previously reinforced with wire. The cells are uncapped with a warmed knife, and the larvae and pupae shaken out onto a clean surface. Since larvae defecate just before pupation, larvae and pupae should be washed in clean water before further processing [3]; (b) By using a small jet of water to remove individual larvae from their cells. This methodology floods one side of the uncapped comb and was reported as reasonably successful. The cells are filled with clean water and then the larvae and pupae are shaken out of them [56]; (c) If the combs are discarded after removal from the hive, they can be squeezed or boiled. The latter works best with new combs. The melted wax hardens at the surface and the larvae sink to the bottom. This method can also have an impact on the organoleptic properties and future usage of the pupae for culinary purposes; (d) A more recent process for the separation is to conduct the process through freezing at low temperature (−20 °C). This procedure preserves the freshness and allows easy breaking up of the comb [6]. Nevertheless, because the honey bee brood and wax freeze too rapidly, only small amounts of material can be processed at a time, and small wax pieces can stick to the brood, making the removal more difficult; (e) The use of liquid nitrogen is also a recent approach and consists of dropping pieces of brood comb into the liquid nitrogen to promote instant freezing. This process increases the time for handling before thawing, but the wax can become too brittle [6].

### 3.3. Storage

Storage conditions and shelf-life considerations impact the product’s quality and safety, which are of particular relevance when the products are for human consumption. Because the larvae and pupae of the *Apis mellifera* are very rich in fat, including monounsaturated fatty acids, they are susceptible to rancidification following oxidation of the fats when in the presence of oxygen. Therefore, these products must be protected from oxidation, which can be performed by freezing and storing them under low temperatures. This can extend their shelf life up 10 months without compromising the nutritional value or changing the organoleptic characteristics [6,57].

### 3.4. Gastronomic Usage

In some countries, such as Mexico, Ecuador, China, Thailand, Senegal, Zambia, and Australia, people eat the eggs, larvae, and pupae of honey bees [6]. In addition, in some Asian countries, honey bee worker or drone pupae (in their white stage) are consumed by humans after pickling or boiling. These pupae are commercialised in canned form in some speciality shops in Europe and the United States. Despite the low market demand in western countries, these are commercialised as value-added products in specific markets [3]. In Asia, an alternative way to process honey bee broods is lyophilisation, and the product is marketed as a powder that has applicability in healthy foods and drinks. When fried, they maintain their shape and become pleasant and crunchy. Regardless of the form, fresh, boiled, or fried, it is reported that honey bee larvae have a rich nutty flavour [58].

Another way to process honey bee larvae is to cover them with chocolate, which are then commercialised as sweet treats. Cans of chocolate-covered honey bee drone larvae may be purchased in some speciality Asian food stores in Europe and the United States [3].

Raw honey bee larvae, when at ambient temperature, are soft and plump. However, when consumed, inside the mouth, they can be cracked by exerting just a slight pressure with the tongue against the palate, thus releasing a mouth-coating liquid from within. Contrarily, raw honey bee pupae at room temperature are a little firmer, which results from their more advanced stage of development, and so they present a higher resistance to pressure than the larvae. Still, they also contain a similar viscous filling inside. When cooked or dried, they tend to retain their shape and are agreeably crunchy, presenting an intense nutty flavour [7].

Citing Daniella Martin, host of the website Girl Meets Bug [59]: “Bee larvae, when sautéed with a little butter and a few drops of honey, taste very much like bacon”, or “I primarily eat drone larvae, which I get from beekeepers…Many beekeepers have a special comb just for drones, which they sometimes use as bait for potential parasites. Periodically, they remove this comb altogether, toss it into the freezer to kill any ‘extras’ like mites, and then either throw it away or feed it to chickens, if they have any. If more people knew how delicious they are, I think the chickens might have to peck elsewhere!” These testimonies emphasise the gastronomic potential of the honey bee drone on one hand and the role of influencers in helping to change minds and incentivise their consumption on the other [60].

As a consequence of the trend of utilising the culling of capped drone broods as part of a natural *Varroa* control strategy by beekeepers, the potential for honey bee drone larvae and pupae to become a commodity is increasing. Nonetheless, the production of honey bee broods is extremely dependent on adequate food availability within colonies. Additionally, honey bee brood production becomes problematic during periods of prolonged dearth, such as during a drought [7].

In some African countries, even those with a cultural tradition of insect consumption, the tourism industry (hotels and restaurants) is looking for new and creative culinary solutions to increase the consumption of insects, just as in other non-insect-eating regions. The utilisation of innovative preparation, production, and presentation of several species of edible insects, with honey bee brood included, is envisaged to increase their uptake. [61].

Chefs worldwide have started to use insects in their culinary preparations, bringing insects to the plane of top gastronomy. These trends highlight their organoleptic or sensory qualities besides their nutritional value [1,62].

When consumed fresh or raw, honey bee larvae have a sweet and fatty taste. If not in a frozen state, the honey bee brood is described as very fragile, thus being susceptible to easy rupture and lost cohesiveness. This is even more evident if the brood has been previously frozen and defrosted [7]. Nevertheless, this textural feature, characterised by its softness, is also a reason for their interesting potential for gastronomic applications. When well-preserved, honey bee broods can attach a surprising and distinctive element to a culinary dish [7]. Table 8 shows some recipes that include honey bee drones and broods in their formulations.

**Figure 1:**
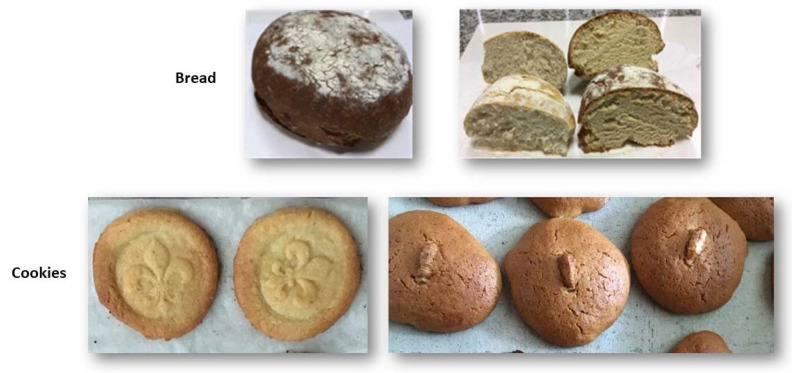
Bakery products that include drone flour.

Drone flour has been used as an innovative food ingredient with different possibilities, facilitating consumption even among consumers who are not traditionally entomophagous. In Viseu, Portugal, drone flour has been developed and attempts have been successfully made to create bakery products that include the drone flour in their formulation (Figure 1). Several studies refer to a higher acceptability of consumers in western countries towards the consumption of foods that contain insects in a more dissimulated way instead of the whole insect [44].

## 4. Conclusions

The *Apis mellifera* brood has a high nutritional value, being particularly rich in protein (including essential amino acids), fat (especially saturated and monounsaturated fatty acids), carbohydrates, vitamins (mainly C and those of the B complex), and minerals (potassium, magnesium, and phosphorous). There are, however, some differences in the proximate composition according to the developmental stage, with increasing content of fat and protein and decrease in carbohydrates from the larval to the pupal stages.

The production of the honey bee brood, particularly drone larvae/pupae, and the techniques used for their removal from the hives are factors that can directly impact the yield as well as quality, so it is important to their usage as a valued added product (or by-product) of the beekeeping sector.

The consumption and purchase of honey bee drone broods as food may be accessible in specialised markets where, for example, ethnic communities consume this type of food. However, in some markets, insects or products produced from insects are not readily accepted because of neophobia and disgust.

## Figures and Tables

**Table 1:** Nutritional composition of honey bee broods [5].

Components	Brood	Components	Brood
**Macro Nutrients (g/kg)**		**Essential Amino Acids (g/kg)**	
Moisture	768	Histidine	2.2
Protein	94	Isoleucine	4.3
Fat	47	Leucine	6.6
Fibre (acid detergent)	3	Lysine	5.8
Fibre (neutral detergent)	2	Methionine	2.0
Ash	8	Phenylalanine	3.3
Carbohydrates	80	Threonine	3.1
**Energy (kcal/kg)**	1119	Tryptophan	0.9
**Energy (kJ/kg)**	4684	Valine	4.9
**Minerals (mg/kg)**		**Nonessential Amino Acids (g/kg)**	
Calcium	138	Alanine	4.5
Phosphorous	1790	Arginine	4.0
Magnesium	211	Aspartic acid	7.6
Sodium	128	Cystine	2.0
Potassium	2690	Glutamic acid	12.9
Chloride	870	Glycine	4.1
Iron	12.9	Proline	5.7
Zinc	16.0	Serine	3.3
Manganese	0.6	Tyrosine	4.1
Copper	4.0	**Others (g/kg)**	
Iodine	<0.1	Taurine ^1^	0.31
Selenium	0.06	Ammonia ^2^	1.9
**Vitamins (mg/kg, or other)**		**Saturated Fatty Acids (g/kg)**	
Beta-carotene (pro-vitamin A)	<0.2	Lauric acid (C12:0)	0.2
Vitamin C	38.0	Myristic acid (C14:0)	1.2
Thiamine (B_1_)	4.1	Palmitic acid (C16:0)	14.7
Riboflavin (B_2_)	9.1	Stearic acid (C18:0)	4.3
Niacin (B_3_)	36.7	Arachidic acid (C20:0)	0.2
Pantothenic acid (B_5_)	11.9	Behenic acid (C22:0)	0.1
Pyridoxine (B_6_)	1.2	**Monounsaturated Fatty Acids (g/kg)**	
Folic acid (B_9_)	<0.06	Palmitoleic acid (C16:1)	0.2
Choline	1684	Oleic acid (C18:1)	18.2
Biotin (B_7_) (μg/kg)	0.23	Eicosenoic acid (C20:1)	0.1
Vitamin B12 (μg/kg)	<1.2	**Polyunsaturated Fatty Acids (g/kg)**	
Vitamin A (IU/kg)	<1000	Linoleic acid (C18:2)	0.3
Vitamin D (IU/kg)	<251	Linolenic acid (C18:3)	0.4
Vitamin E (IU/kg)	<5.0	Eicosadienoic acid (C20:2)	0.1

^1^ Taurine is a conditional amino acid resulting from cysteine metabolism [33]. ^2^ Ammonia results from glutamine and alanine metabolism [34].

**Table 2:** Nutritional composition of larvae and pupae of *Apis mellifera ligustica* worker bees [36].

Components		Larvae	Pupae
Macro Components ^1^	Moisture (g/100 g)	74.4	79.3
	Protein (g/100 g d.m.)	35.3	45.9
	Fat (g/100 g d.m.)	14.5	16.0
	Ash (g/100 g d.m.)	4.1	3.8
	Carbohydrates (g/100 g d.m.)	46.1	34.3
Energy (kcal/100 g d.m.)		455.8	465.0
Essential Amino Acids (g/100 g d.m.)	Valine	1.7	2.4
	Isoleucine	1.6	2.3
	Leucine	2.5	3.2
	Lysine	1.9	3.0
	Tyrosine	1.5	2.0
	Threonine	1.6	1.9
	Phenylalanine	0.2	0.2
	Histidine	0.7	1.1
	Tryptophan	Not detected	Not detected
Nonessential Amino Acids (g/100 g d.m.)	Arginine	1.6	2.3
	Aspartic acid	2.6	3.5
	Serine	1.4	2.0
	Glutamic acid	5.0	8.4
	Glycine	1.4	2.5
	Alamine	1.6	2.9
	Cysteine	0.3	0.4
Saturated Fatty Acids (mg/100 g d.m.)	Capric acid (C10:0)	Not detected	Not detected
	Lauric acid (C12:0)	15.5	24.6
	Myristic acid (C14:0)	116.6	157.5
	Palmitic acid (C16:0)	1844.0	1942.2
	Stearic acid (C18:0)	584.9	696.8
Monounsaturated Fatty Acids (mg/100 g d.m.)	Hexadecenoic acid (C16:1)	35.1	31.1
	Oleic acid (C18:1)	2346.1	2632.1
	Eicosenoic acid (C20:1)	Not detected	Not detected
Polyunsaturated Fatty Acids (mg/100 g d.m.)	Linoleic acid (C18:2)	Not detected	Not detected
Minerals (mg/100 g)	Calcium	84.9	97.0
	Magnesium	177.0	193.9
	Sodium	59.4	60.8
	Potassium	1871.9	2207.3
	Iron	13.3	15.3
	Zinc	11.6	11.7
	Copper	3.6	3.7
	Manganese	1.2	0.7
	Phosphorous	782.5	900.0

^1^ While moisture was expressed in wet basis (g/100 g sample), all other values are in dry basis (g/100 g dry matter (d.m.)).

**Table 3:** Nutritional composition of larvae and pupae of *Apis mellifera* [37].

Components		Larvae	Pupae
Macro Components (%)	Protein	19.0	24.6–26.6
	Fat	28.1	19.1–21.1
	Ash	2.8	3.5–3.2
	Carbohydrates	50.1	50.8–51.1
Saturated Fatty Acids (%)	Myristic acid (C14:0)	3.0	2.4–2.7
	Palmitic acid (C16:0)	34.5	28.7–31.2
	Stearic acid (C18:0)	10.4	11.3–12.5
	Arachidic acid (C20:0)	1.0	1.8
	Behenic acid (C22:0)	1.0	2.0–2.1
Unsaturated Fatty Acids (%)	Oleic acid (C18:1)	45.9	46.6–48.7
	Linoleic acid (C18:2)	1.5	2.2–2.3
	Linolenic acid (C18:3)	2.6	2.2–2.3

**Table 4:** Amino acid profile of drone pupae of *Apis mellifera* subspecies [38].

Amino Acids (g/100 g d.m.) ^1^	*Apis mellifera mellifera* ^2^	*Apis mellifera carnica* ^2^	*Apis mellifera ligustica* ^2^	*Apis mellifera buckfast* ^2^
**Essential AA**				
Valine	1.9–2.4	1.8–2.5	2.6–3.0	2.9–3.0
Isoleucine	1.6–2.2	1.6–2.2	2.1–2.4	2.4–2.6
Leucine	2.7–3.5	2.6–3.6	3.5–4.1	4.0–4.3
Lysine	2.4–3.1	2.3–3.2	3.0–3.5	3.5–3.7
Threonine	1.4–1.7	1.3–1.7	1.9	1.6–1.9
Histidine	0.8–1.1	0.8–1.1	0.9–1.1	1.2–1.3
Sulphur-containing AA	1.0–1.8	0.6–1.1	0.4–0.7	1.4–1.5
Aromatic AA	3.0–3.9	3.0–3.8	4.0–4.8	4.6–4.9
**Nonessential AA**				
Arginine	1.7–2.3	1.7–2.3	2.2–2.6	2.2–2.5
Aspartic acid	2.4–3.0	2.4–2.8	2.5–2.7	3.2
Serine	1.4–2.0	1.4–1.9	1.8–2.1	2.0–2.4
Glutamic acid	6.6–8.1	6.3–7.4	10.0–10.6	7.9–8.8
Glycine	1.6–2.4	1.5–2.6	2.1–2.8	2.3–2.7
Alamine	1.5–2.5	1.5–2.9	2.6–3.4	2.4–2.9
Cysteine	2.8–3.6	2.4–3.7	3.0–3.6	1.6–1.5

^1^ Expressed in dry basis (g/100 g dry matter). ^2^ Range of values considering the different stages (prepupal, early pupal, late pupal).

**Table 5:** Fatty acid profile of drone pupae of *Apis mellifera* subspecies [38].

Fatty Acids (mg/100 g d.m.) ^1^	*Apis mellifera mellifera* ^2^	*Apis mellifera carnica* ^2^	*Apis mellifera ligustica* ^2^	*Apis mellifera buckfast* ^2^
**Saturated FA**				
Capric acid (C10:0)	0–1.8	2.0	n.d.	n.d.
Lauric acid (C12:0)	20.9–26.0	27.6–29.8	32.5–33.4	26.0–31.4
Myristic acid (C14:0)	284.1–354.0	234.7–379.3	258.1–333.1	359.5–365.5
Palmitic acid (C16:0)	3804–4848	3307–4699	3571–4518	4810–4879
Margaric acid (C17:0)	4.3–4.5	4.1–4.2	n.d.	n.d.
Stearic acid (C18:0)	1181–1260	1207–1363	1267–1357	1110–1303
Arachidic acid (C20:0)	45.1–67.7	46.8–72.4	120.6–145.8	0–56.2
Behenic acid (C22:0)	16.9–27.6	16.0–30.3	14.4–23.3	n.d.
Lignoceric acid (C24:0)	n.d.	n.d.	39.2–42.6	n.d.
***Subtotal***	***5397–6484***	***4885–6476***	***5341–6414***	***6306–6635***
**Monounsaturated FA**				
Myristoleic acid (C14:1)	2.4–3.1	0–2.4	n.d.	n.d.
Palmitoleic acid (C16:1)	56.1–72.3	47.9–55.4	47.7–48.3	51.9–56.4
Elaidic acid (C18:1t)	n.d.	n.d.	0–6.8	n.d.
Oleic acid (C18:1)	4197–4579	4316–4771	4412–4903	4720–5105
Eicosenoic acid (C20:1)	6.6–8.5	7.3–9.1	8.7–10.4	n.d.
***Subtotal***	***4264–4655***	***4373–4832***	***4471–4966***	***4777–5156***
**Polyunsaturated FA**				
Linolelaidic acid (C18:2t)	21.3–22.2	10.2–17.3	n.d.	n.d.
Linoleic acid (C18:2)	31.3–56.8	36.3–49.0	22.8–30.7	0–67.9
Linolenic acid (C18:3)	77.4–118.7	151.9–154.1	61.2–83.2	n.d.
Mead acid (C20:3)	n.d.	0–1.8	n.d.	n.d.
Docosadienoic acid (C22:2)	13.0–19.4	14.9–26.2	15.2–17.2	n.d.
Eicosapentaenoic acid (C20:5)	6.5–7.4	3.9–7.3	n.d.	n.d.
***Subtotal***	***149.4–223.8***	***228.6–242.8***	***99.2–131.2***	***0–67.9***
**Total**	**9885–11,303**	**9502–11,547**	**9943–11,479**	**11,082–11,859**

n.d. = not detected. ^1^ Expressed in dry basis (mg/100 g dry matter). ^2^ Range of values considering the different stages (prepupal, early pupal, late pupal).

**Table 6:** Minerals of drone pupae of *Apis mellifera* subspecies [38].

Minerals (mg/100 g d.m.) ^1^	*Apis mellifera mellifera* ^2^	*Apis mellifera carnica* ^2^	*Apis mellifera ligustica* ^2^	*Apis mellifera buckfast* ^2^
Calcium (Ca)	39.3–43.3	34.0–46.1	43.7–49.3	34.2–38.7
Magnesium (Mg)	70.2–85.8	65.9–88.4	82.9–95.0	68.1–81.9
Sodium (Na)	8.1–9.9	7.0–10.3	7.3–8.5	30.1–38.0
Potassium (K)	1080–1342	1048–1401	544.6–643.1	891.1–1102.0
Phosphorus (Ph)	673.5–812.3	651.7–869.2	774.0–892.4	686.9–802.6
Iron (Fe)	4.7–5.7	5.6–6.1	4.9–5.7	5.6–6.0
Zinc (Zn)	4.4–5.5	4.8–6.0	5.3–5.9	5.1–6.0
Copper (Cu)	1.5–1.9	1.6–2.0	1.8–1.9	0.1–0.4

^1^ Expressed in dry basis (mg/100 g dry matter (d.m.)). ^2^ Range of values considering the different stages (prepupal, early pupal, late pupal).

**Table 7:** Vitamins of *Apis mellifera* brood [38].

Vitamins (μg/100 g) ^1^	Worker Larvae (Day 9)	Worker Pupae (Day 19)	Brood	Mature Larvae	Pupae	Drone Pupae
Vitamin A	1.32	7.41	<1 ^2^	89–119 ^2^	49.3–53.3 ^2^	Not detected
Vitamin B_1_ (Thiamine)	0.94	3.27	410	—	—	1550
Vitamin B_2_ (Riboflavin)	—	251	910	—	—	2930
Vitamin B_3_ (Niacin)	—	—	3670	—	—	—
Vitamin B_5_ (Pantothenic acid)	—	—	1190	—	—	—
Vitamin B_6_ (Pyridoxine)	—	—	120	—	—	—
Vitamin B_7_ (Biotin)	—	—	0.023	—	—	—
Vitamin B_9_ (Folic acid)	—	—	<0.006	—	—	—
Vitamin B_12_	—	—	<0.12	—	—	—
Vitamin C	4020	4350	3800	—	—	—
Vitamin D	390	410	<0.25 ^2^	6130–7430 ^2^	5070–5260 ^2^	Not detected
Vitamin E	0.87	1.10	<0.005 ^2^	—	—	6060
Choline	—	—	168.4	—	—	—

^1^ Unless other units are presented. ^2^ IU/g.

**Table 8:** Gastronomic preparations which include *Apis mellifera* brood.

Gastronomic Preparations		Links (accessed on 20 May 2022)
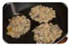	Bee Larvae Fritters	https://www.youtube.com/watch?v=vjDfZjtfqTc
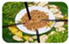	Fermented Bee Larvae with Vegetables	http://www.thanhniennews.com/arts-culture/fermented-bee-larvae-a-gift-of-the-mekong-deltas-cajuput-forest-58938.html
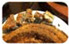	Grilled Bee Larvae: A Cambodian Street Snack	https://www.youtube.com/watch?v=2h7F2Ca6szg
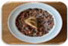	Honeybee Granola	https://www.bugsfeed.com/honeybee_granola
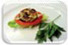	Sandwich Prepared with Bee Larvae	https://www.bugsfeed.com/bee_lt_sandwich
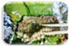	Grilled Bee Larvae with Honeycomb	https://www.streetfoodguy.com/grilled-bee-larvae-with-honeycomb/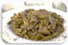	Vietnamese Fried Bee Pupae	http://kyspeaks.com/2007/08/27/ky-eats-fried-bee-pupae-at-vietnam/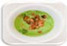	Peas and Bees	https://www.bugsfeed.com/peas_bees
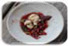	Baby Bee Ceviche, with Bee larvae	https://www.bugsfeed.com/baby_bee_ceviche
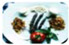	Honeybee Larvae Appetizers	https://www.fao.org/3/w0076e/w0076e19.htm
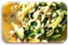	Thai Cooking-Bee Eggs and Bee Larvae	https://www.youtube.com/watch?v=G3SAjesHYpk

## Data Availability

There are no data referring to this review article.
